# Perspective: Of Mice and Men – How Widespread Is Adult Neurogenesis?

**DOI:** 10.3389/fnins.2019.00923

**Published:** 2019-08-29

**Authors:** David Petrik, Juan M. Encinas

**Affiliations:** ^1^School of Biosciences, Cardiff University, Cardiff, United Kingdom; ^2^Institute of Stem Cell Research, Helmholtz Zentrum München, Munich, Germany; ^3^Department of Physiological Genomics, Ludwig Maximilian University of Munich, Munich, Germany; ^4^Laboratory of Neural Stem Cells and Neurogenesis, Achucarro Basque Center for Neuroscience, Leioa, Spain; ^5^IKERBASQUE, The Basque Foundation for Science, Bilbao, Spain; ^6^Department of Neurosciences, University of the Basque Country (UPV/EHU), Leioa, Spain

**Keywords:** neuroscience, neural stem/ progenitor cells, hippocampus, human neurogenesis, adult neurogenesis

## Abstract

These are exciting times for research on adult hippocampal neurogenesis (AHN). Debate and controversy regarding the existence of generation of new neurons in the adult, and even diseased human brain flourishes as articles against and in favor accumulate. Adult neurogenesis in the human brain is a phenomenon that does not share the qualities of quantum mechanics. The scientific community should agree that human AHN exists or does not, but not both at the same time. In this commentary, we discuss the latest research articles about hAHN and what their findings imply for the neurogenesis field.

“The best-laid schemes o’ mice an’ men gang aft agley” (The best laid schemes of mice and men go often askew)

Robert Burns (1785)

It is common that new concepts are doubted and re-doubted. We already overcame the once disbelieve in the existence of adult neurogenesis in the mammalian brain. However, a new controversy arose recently about the existence of human AHN (hAHN), the process of generating adult-born neurons from neural stem cells (NSCs). While it is not the first time that the existence of adult neurogenesis has been discredited (Rakic, 1985), the findings that adult neurogenesis may not exist in adult human hippocampus (Cipriani et al., 2018; Sorrells et al., 2018) come at a time when research on adult neurogenesis constitutes a major field in neurosciences due to the importance to the functions (memory, learning and mood control) associated with this phenomenon in animal models (Eisch and Petrik, 2012). The findings by Cipriani et al. (2018) and Sorrells et al. (2018) (Figure 1) are in direct conflict with another three major recent studies demonstrating hAHN (Boldrini et al., 2018; Moreno-Jiménez et al., 2019; Tobin et al., 2019) following the path of previous reports (Fahrner et al., 2007; Knoth et al., 2010). This most recent controversy does not only stir up the research community but also examines its conceptual and structural complexion.

**FIGURE 1:**
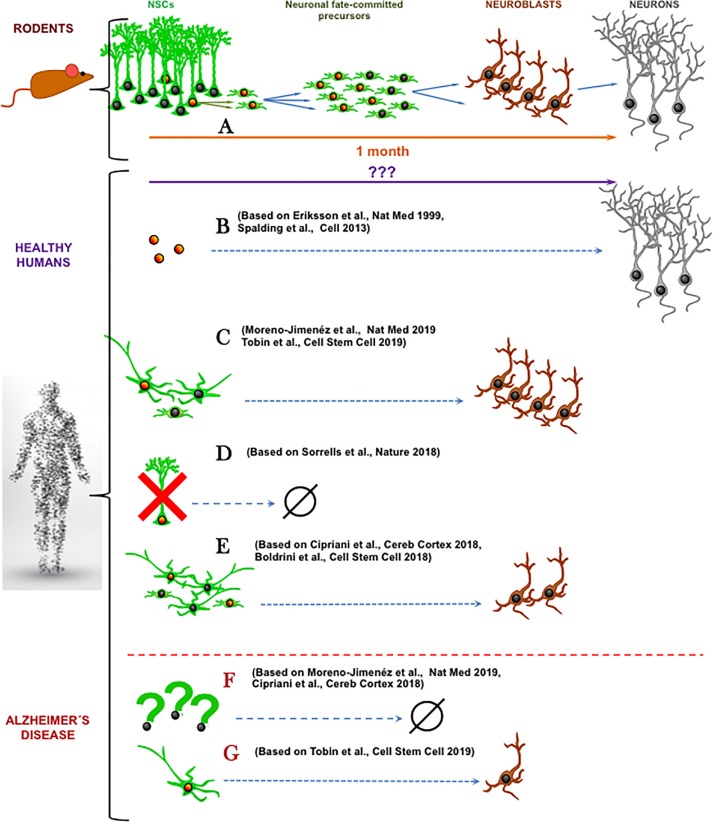
Schematic summary of recent research articles on human adult hippocampal neurogenesis. **(A)** In mice, a population of well-characterized neural stem cells (NSCs) generates neuronal-fate committed precursors that amplify their numbers through cell divisions and then differentiate into neuroblasts that maturate into neurons. **(B)** In healthy humans, cell-division dependent neurogenesis has been reported using radioactive carbon-based cell-birth dating (Spalding et al., 2013) and BrdU incorporation (Eriksson et al., 1998). **(C)** The abundant presence of both neural precursor like-cells and immature neurons (Tobin et al., 2019) or immature neurons (Moreno-Jiménez et al., 2019) has been shown, coexisting with cell division markers. **(D)** Total absence of cell proliferation, neural precursor like-cells and immature neurons has been demonstrated in the adult hippocampus (Sorrells et al., 2018). **(E)** Present, but reduced number of neural progenitor-like cells and immature neurons has been reported (Cipriani et al., 2018; Boldrini et al., 2018). In Alzheimer’s disease, neural precursor like-cells and immature neurons are greatly reduced (Cipriani et al., 2018; Moreno-Jiménez et al., 2019) **(F)** or still exist in more prominent cell populations (Tobin et al., 2019) **(G)**. Orange nuclei indicate cell division while nuclei indicate not proliferative state. (Human shape Designed Freepik-Vilmosvarga).

hAHN has been proposed to exist using a plethora of techniques ranging from immunohistochemistry for native or synthetic markers of proliferation (Eriksson et al., 1998), cell markers of neuroblasts and immature neurons (Knoth et al., 2010), to unique radioactive carbon-based cell-birth dating (Spalding et al., 2013) and non-invasive imaging approaches (Manganas et al., 2007). All of these, however, focused on the hippocampus, whereas human neurogenesis in the walls of lateral ventricles has remained far less studied. Thus, when a second wave of controversy on adult neurogenesis in humans had appeared, driven by the findings of Alvarez-Buylla lab (Sanai et al., 2011), most of the adult neurogenesis researchers were (and still are) disconcerted, largely because ventricular neurogenesis lies outside of the predominant hippocampus-focused interest. The findings of Sanai et al. (2011) that adult humans do not show neurogenesis derived from the subventricular zone of the lateral ventricles contradict articles claiming its presence (Curtis et al., 2007; Ernst et al., 2014). This report that the subventricular NSCs quickly disappear from human brain during infancy should have been perceived with a greater urgency that similar studies will be published about hAHN. The lack of reaction in the research field says a lot about its structure and the way it sees its own subject of study. Such hippocampus-heavy tendency can be further appreciated in the fields’ disinterest in so called non-canonical adult neurogenesis in the hypothalamus, where unique adult NSCs generate diet-responsive adult born neurons (Yoo and Blackshaw, 2018). Interestingly, hypothalamic neurogenesis regulates ventricular neurogenesis (Paul et al., 2017) and therefore now more than ever it is important to investigate if the hypothalamic neurogenesis also exists in adult humans and to what degree (Pellegrino et al., 2018).

Adult neurogenesis has been confirmed in the majority of species of terrestrial mammals, but it seems to be absent in cetaceans, reviewed in detail in Amrein (2015), Patzke et al. (2015), Lipp and Bonfanti (2016). Markers for cell proliferation, stem cells and immature neurons were identified in adult hippocampus of mammals with small, lissencephalic brains such as rodents, but also in large, gyrencephalic brains of phylogenetically distant species such as the cows (Rodriguez-Perez et al., 2003), the African elephants (Patzke et al., 2014), or the dogs (Hwang et al., 2007). Furthermore, adult neurogenesis has been found in hippocampus of various different primate species including marmosets (Bunk et al., 2011), lemurs (Fasemore et al., 2018), macaques (Gould et al., 2001; Jabes et al., 2010) and baboons, where adult neurogenesis is required for the antidepressant action (Perera et al., 2011; Wu et al., 2014). The prevalence of adult neurogenesis in primates suggests that it should be found also in humans. However, phylogenetics may not be the most reliable predictor of adult neurogenesis existence even in related taxonomic ranks. For example, some species of bats do have active neurogenesis, while others do not (Amrein, 2015). This could be caused by natural differences in closely related taxons or it could stem from technical reasons, which may not be the case for well prepared specimens of bat brain (Amrein et al., 2007) but could apply to more complicated autopsies of human tissue. Indeed, some native cell markers for neurogenesis are sensitive to fast degradation and specific tissue fixation, which can be the most likely factor to explain the disagreement in the results regarding the human data (Lucassen et al., 2019).

The immunohistochemical detection of individual cell markers may not support the existence of adult neurogenesis, however, their combination could (as summarized elsewhere (Kempermann et al., 2018). For example, Moreno-Jiménez et al. (2019) reported PSA-NCAM or doublecortin (DCX+) positive cells in human hippocampus as an evidence of adult neurogenesis, because these markers label neuroblasts or immature adult-born neurons in mice (Kempermann et al., 2004) and other mammals. On the other hand, Sorrells et al. (2018) reported a lack of DCX+ and PSA-NCAM+ neurons as well as the sharp decline of proliferating cells labeled by Ki67, the endogenous marker of cell cycle. Because Moreno-Jiménez et al. (2019) did not stained for proliferation markers, an argument could be made that the observed DCX+ neurons are not a direct product of adult neurogenesis but rather a unique subset of neurons expressing markers associated with neurogenesis. However, both immature neurons and proliferating Ki67+ or PCNA+ cells or proliferating Ki67+ Nestin+ putative progenitor cells have been demonstrated in the other most recent studies (Boldrini et al., 2018; Tobin et al., 2019) or in previous studies on adult hippocampus neurogenesis in humans that used either endogenous (Liu et al., 2008; Knoth et al., 2010; Dennis et al., 2016; Mathews et al., 2017) or synthetic markers of proliferation (Eriksson et al., 1998; Ernst et al., 2014).

In our opinion two major questions arise from these recent data. First, how do we actually define adult neurogenesis? Based on the literature consensus, adult neurogenesis is the generation, through cell division of neural progenitors, of new neuronal fate-committed precursors that undergo a process of neuronal differentiation and maturation (Figure 1A). Second, what is needed in terms of biomarker expression to accept this definition of adult neurogenesis? Expression of DCX and or PSA-NCAM may not be sufficient. The existence of very slowly maturing neurons which maintain the expression of these immature markers but were actually generated during development has been demonstrated in the brain of rodents and sheep (Piumatti et al., 2018; La Rosa et al., 2019). This process that represents another fascinating form of brain plasticity supports the argument that exploring cell divisions should be a requisite for confirmation of adult neurogenesis in humans. On the other hand, presence of cell division together with the presence of neuroblasts or immature neurons may not be sufficient criteria for claiming neurogenesis. Even in the neurogenic niches, there are other actively dividing cell types such as astrocytes, microglia, pericytes, endothelial cells and oligodendrocyte progenitors (OPCs). Some of these cell types share specific cell markers with neural precursors. For example, nestin is present in OPCs and perycites (Encinas et al., 2011) and Sox2 is expressed in all astrocytes in the hippocampus (Komitova and Eriksson, 2004). This shared expression of certain cell markers is of special relevance in aging. One of the hallmarks of astrocytes in the aged brain is the gradual acquisition of a reactive-like and even proliferative phenotype (Clarke et al., 2018), which is further characterized by the expression of nestin. Thus, expression of nestin and Sox2 may not constitute a valid marker combination to exclusively identify neural progenitors. Instead, the stem cells and progenitors should be described by more recent specific biomarkers such as Lunatic fringe (*Lfng)* (Semerci et al., 2017) or the lysophosphatidic acid receptor 1 (*LPAR1*) (Walker et al., 2016) and by exclusion of expression of S100ß, a marker of mature astrocytes. Finally, another strategy could be utilized to strengthen the conclusions about hAHN – a correlation between levels of cell division in progenitors and levels of DCX or PSA-NCAM in immature neurons. Even though correlation does not imply causation, a positive correlation would point toward the existence of a neurogenic cascade, adding up to the earlier works by Spalding et al. (2013) and Eriksson et al. (1998). These works suggest the existence of neurogenesis in the adult human brain (Figure 1B) by detecting in neurons markers that would have been incorporated, arguably (see Duque and Spector, 2019. for critical technical analysis), of those cells only through mitosis.

This newest controversy on the existence of adult neurogenesis in human hippocampus highlights other aspects than just definition of cellular stages by specific markers. First, there is the issue of time (reviewed in detail in Snyder, 2019). Mice live about 50 times shorter than humans, yet their adult neurogenesis declines more rapidly with age, whereas human neurogenesis could persist for up to 80 decades (Knoth et al., 2010; Moreno-Jiménez et al., 2019). If hAHN exists, what are the mechanisms that allow humans to maintain active putative neural progenitors for so much longer? What could be the key molecular determinants for such long-term cellular “stemness”? Importantly, when we look at studies using human samples, opposite results on the existence of neural progenitors emerge. Neural progenitors are harder to be determined in human samples due to limited technical toolbox as explained above and when addressed, opposite results have been found. While Sorrells et al. (2018) report a drastic reduction of neural stem and progenitor-like cells that would thus explain the absence of adult neurogenesis, Boldrini et al. (2018) and Tobin et al. (2019) report their abundant existence. Half way, Cipriani et al. (2018) showed persistence of neural stem and progenitor-like cells in the adult brain, but absence of actual neurogenesis. In any case, the properties of these neural progenitors are yet poorly studied and could be different from those of the mouse. For instance, according to the published data, in human samples putative NSCs would have to have a more stellate morphology than a radial one (Boldrini et al., 2018; Cipriani et al., 2018; Tobin et al., 2019) (Figures 1C–E). Second, the most recent studies by Moreno-Jiménez et al. (2019) and Tobin et al. (2019) again confirm abundant adult neurogenesis, or at least the abundant presence of immature neurons, in healthy human hippocampus but limited or absent neurogenesis in patients with Alzheimer’s Disease (AD) (Figure 1F). This finding that AD alters adult neurogenesis does not only underscore the necessity of proper triage of diseased tissue specimens in human studies but is in line with conclusions from AD mouse models (Hamilton et al., 2015). However, as with many compounded topics such as modeling of the AD in rodents, it is apparent that the small, lissencephalic brains of mice may not be the best proxy for the large and complex human brains (Jankowsky and Zheng, 2017). To put it bluntly: mice are not small humans. And yet, sort of automatic assumptions are drawn from rodent models to functional implications in humans. As commented before, it could be that the neuronal maturation process is much slower in humans and that the ratio between cell proliferation and maturing neurons is much weaker than in mice. Very slowly maturing neurons would read out as an apparent higher-than-real amount of neurogenesis (which implies birth of neurons). On the other hand, if humans lack adult neurogenesis, how would all the adult neurogenesis-dependent brain functions described in rodents operate in humans without adult neurogenesis? And what are the reasons why humans have diverged in evolution from other primates that contain AHN?

We should use the current debate to re-evaluate the *status quo* of the neurogenesis field with respect to the laboratory models, quality controls and theoretical concepts to move the topic and the field forward. In other words, humans are not large mice; disease, metabolism and life style can negatively affect the tissue and lead us to inaccurate conclusion; and molecular mechanisms driving NSCs in hippocampus (Petrik et al., 2015) may not be the same in the other neurogenic niches (Ninkovic et al., 2013). Furthermore, this recent surge in interest in human adult neurogenesis should be employed to re-evaluate if adult neurogenesis is prevalent in parts of human brain other than the hippocampus. How well established is the fact that adult neurogenesis is actually absent in the ventricular system of the adult human brain? It is possible that the evolutionary pressures for greater complexity in human brain did not strip it from the subventricular neurogenesis (Sanai et al., 2011), but rather made neurogenesis more prevalent in regions of the central nervous system where we have not yet looked in both physiological and pathophysiological conditions? In conclusion, first we have to stipulate what technical criteria are essential to identify adult neurogenesis. And then we should not only ask whether adult neurogenesis does exist in the human brain or not, but we should also ask whether it occurs as a process similar to rodent neurogenesis, or whether it is more wide-spread than we originally thought.

## Author Contributions

Both authors contributed equally to the writing of this manuscript.

## Conflict of Interest Statement

The authors declare that the research was conducted in the absence of any commercial or financial relationships that could be construed as a potential conflict of interest.
